# In-Vivo Characterization of Healthy Retinal Pigment Epithelium and Photoreceptor Cells from AO-(T)FI Imaging

**DOI:** 10.3390/vision9040091

**Published:** 2025-11-01

**Authors:** Sohrab Ferdowsi, Leila Sara Eppenberger, Safa Mohanna, Oliver Pfäffli, Christoph Amstutz, Lucas M. Bachmann, Michael A. Thiel, Martin K. Schmid

**Affiliations:** 1 EarlySight SA, 1202 Geneva, Switzerland; 2 Department of Radiology and Medical Informatics, Faculty of Medicine, University of Geneva, 1202 Geneva, Switzerland; 3 Eye Clinic, Cantonal Hospital of Lucerne, 6000 Lucerne, Switzerland; 4 Department of Ophthalmology, Inselspital, University Hospital, 3010 Bern, Switzerland; 5 Singapore Eye Research Institute, Singapore 169856, Singapore; 6 Eye Clinic, Cantonal Hospital of Winterthur, 8400 Winterthur, Switzerland; 7 Medignition A.G., 8004 Zurich, Switzerland; 8 Epidemiology, Biostatistics & Prevention Institute (EBPI), University of Zurich, 8001 Zurich, Switzerland; 9 Faculty of Health Sciences and Medicine, University of Lucerne, 6005 Lucerne, Switzerland

**Keywords:** adaptive optics, high resolution retinal imaging, in-vivo clinical eye research, retinal pigment epithelium, photoreceptors, retinal diseases

## Abstract

We provide an automated characterization of human retinal cells, i.e., RPE’s based on the non-invasive AO-TFI retinal imaging and PR’s based on the non-invasive AO-FI retinal imaging on a large-scale study involving 171 confirmed healthy eyes from 104 participants of 23 to 80 years old. Comprehensive standard checkups based on SD-OCT and Fondus imaging modalities were carried out by Ophthalmologists from the Luzerner Kantonsspital (LUKS) to confirm the absence of retinal pathologies. AO imaging imaging was performed using the Cellularis^®^ device and each eye was imaged at various retinal eccentricities. The images were automatically segmented using a dedicated software and RPE and PR cells were identified and morphometric characterizations, such as cell density and area were computed. The results were stratified based on various criteria, such as age, retinal eccentricity, visual acuity, etc. The automatic segmentation was validated independently on a held-out set by five trained medical students not involved in this study. We plotted cell density variations as a function of eccentricity from the fovea along both nasal and temporal directions. For RPE cells, no consistent trend in density was observed between 0° to 9° eccentricity, contrasting with established histological literature demonstrating foveal density peaks. In contrast, PR cell density showed a clear decrease from 2.5° to 9°. RPE cell density declined linearly with age, whereas no age-related pattern was detected for PR cell density. On average, RPE cell density was found to be ≈6313 cells/mm^2^ (±σ=757), while the average PR cell density was calculated as ≈10,207 cells/mm^2^ (±σ=1273).

## 1. Introduction

While the Photoreceptor cells (PR’s) are responsible for detecting light and initiating visual processing of the retina, the Retinal Pigment Epithelium (RPE) cells play a critical supporting role as they perform nutrition support, ion transfer, immunological, waste management, as well as secretory roles for various retinal layers [1,2,3,4].

Dysfunction within any of these two specialized cell layers can be associated with retinal degeneration [5,6,7] and, hence, lead to various retinal diseases [8]. Conditions such as Age-Related Macular degeneration (AMD) or Retinitis Pigmentosa are characterized by the progressive loss of PR cells, often triggered or exacerbated by impaired RPE functioning [9,10].

To better understand retinal diseases and their progression, particularly as motivated by recent therapeutic options for these retinal disorders [11,12,13], it is therefore crucial to monitor the retina at the cellular level.

Despite great success and wide adoption of standard retinal imaging modalities such as high-resolution spectral domain optical coherence tomography (SD-OCT) and fundus autofluorescence (AF), visualizing the RPE at the cellular level remains challenging ([14,15,16,17,18,19,20,21,22]).

Over the past two decades, the development and commercialization of adaptive optics (AO) has enabled in vivo observation of PR’s using both SD-OCT and scanning laser ophthalmoscopy ([23]). Consequently, researchers have tried to refine AO technologies to be able to image the RPE ([24,25]), with several examples achieving some success within research settings ([26,27]). These techniques, however, remain as research prototypes and their efficacy in clinical settings is hindered by prolonged acquisition times and limited fields of view.

In 2020, Laforest et al. showed that by combining transscleral flood illumination consisting of dual oblique beams and with an AO fundus camera, in vivo images of human RPE, PR and nerve fiber layer with a high signal-to-noise ratio was possible [28]. Near-infrared light (wavelength = 850 nm) is directed through the nasal and temporal sclera into the eye by two light-emitting diodes positioned laterally. After being scattered from the deeper cell layers of the retinal RPE layer, light exits the pupil and is collected by the optical system. The Adaptive Optics Transscleral Flood Illumination (AO-TFI) of [28] then became established under the Cellularis^®^ prototype and later commercialized as Cellularis^®^ Discovery device as an in-vivo ophthalmic camera.

Currently, our understanding of the retinal cells is based partly on ex-vivo studies on cadaver donors eyes. Various studies have tried to characterize main RPE/PR morphological descriptors such as cell density and area. For example, studies [29,30,31] report results based on 20, 19 and 17 donated eyes, respectively and each under different experimental settings.

Apart from the ex-vivo histological studies, in-vivo imaging has been achieved thanks to the adoption of Adaptive Optics (AO) technology. The PR layer was the first to have been imaged using this technology at the cellular level with early attempts such as [32,33], or more recent works like [34]. The study of [35] achieves rod and cone characterizations based on AO-SLO imaging of 24 healthy eyes from 21 participants. The recent study [36] reports PR characterization results on 36 healthy volunteers based on the RTX1 device (Imagine Eyes, Orsay, France).

In-vivo imaging of the RPE cells, however, is more challenging, since the RPE layer is located below the highly reflective PR layer. Attempts at the characterization of the RPE layer, however, are very recent. The work of [37] is a visual case study where they manage to image RPE structures on 4 out of the 7 healthy participants using AO-SLO. The work of [38] involved 29 participants where an earlier prototype of the Cellularis device was used for imaging of the RPE layer.

The study of [39] based on AOSLO imaging reports results of 10 healthy participants estimating the mean RPE density to be around 6026 cells/mm^2^ at the foveal region and decreasing to 4552 cells/mm^2^ and 3757 cells/mm^2^ at the at the temporal and nasal sides, respectively.

Finally, the recent study of [40] uses a custom FDML-AO-OCT device to image and characterize the PR-RPE complex in 11 healthy and young participants. They estimate the average foveal RPE density to be 7335 (±681) cells/mm^2^, while they provide detailed comparisons of their results with the existing literature including ex-vivo studies. The reader is referred to their paper for a more detailed and recent review of the literature in RPE and PR characterization attempts.

As will be described next, our study in this paper provides detailed morphometric characterization of both RPE and PR cells with in-vivo imaging of 171 healthy eyes from 104 participants imaged using the AO-TFI technology. This morphometric characterization consists of cell density (cells/mm^2^), area (μm^2^), perimeter (μm), as well as the number of neighbors in Voronoi tessellations. Our aim is to establish a large-scale, in-vivo normative database of Photoreceptor and RPE morphometry in healthy human eyes across the entire adult life-span using AO-TFI and automated segmentation. To the best of our knowledge, this is the largest study reporting results on RPE or PR, and also within the in-vivo or ex-vivo literature.

As a note on technological limitations of the imaging technology used, for the PR layer, we excluded foveal eccentricities between −2.5° to 2.5° from our measurements, since optical resolution was limited at this central foveal region of cone Photoreceptors. Moreover, in our AO-TFI configuration, the optical resolution and reflectance contrast are optimized for cone visualization, as rod photoreceptors fall below the system’s detection threshold. Additionally, the origin of the coordinate system which is supposed to be the fovea is approximative in our study and was based on a fixation target where the participants were supposed to gaze at during the imaging.

## 2. Materials and Methods

### 2.1. Clinical Study

#### 2.1.1. Design

This study is part of a cross-sectional clinical study that was run at the Eye Clinic of the Kantonsspital Lucerne between May 2021 and October 2022 in compliance with the Declaration of Helsinki and ISO 14155 [41]. The protocol (ClinicalTrials.gov: NCT04912622; kofam.ch, SNCTP000004502) was approved by the regional ethics committee (BASEC: EKNZ2020-02454) and the Swiss Agency for Therapeutic Products (Swissmedic).

#### 2.1.2. Recruitment

Participants with no self-reported history of retinal disease were enrolled. Recruitment took place during pre-cataract examinations and additionally included accompanying relatives of patients as well as volunteer staff from the Eye Clinic. All participants provided written informed consent prior to enrollment.

#### 2.1.3. Ocular Examination and Image Acquisition

Demographics, visual acuity, multi-modal imaging data, including SD-OCT (Spectralis^®^, Heidelberg Engineering, Heidelberg, Germany), and adverse events (AEs) during image acquisition, were collected for all participants and all eyes. Prior to AO imaging with Cellularis^®^ (prototype version 2.0, EarlySight, Geneva, Switzerland), axial length (AL) was measured by optical biometry (IOL-Master 700^®^, Zeiss, Germany; Optical Biometer OA-2000, Tomey, Japan). Two trained personnel evaluated the SD-OCTs and autofluorescence images. Participants who, despite self-reporting no ocular disease at enrollment, were found to have retinal abnormalities upon examination were excluded. All participants underwent a comprehensive ophthalmic examination prior to imaging. Inclusion criteria required a clear crystalline lens without significant opacities (Lens Opacities Classification System III grade ≤1), and no history of cataract surgery or intraocular lens implantation. Any subject with more advanced lens changes or pseudophakia was excluded from the study.

**Figure 1:**
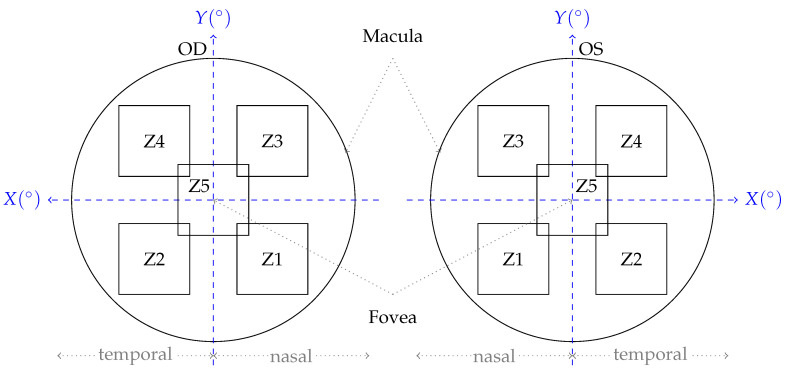
AO-TFI image acquisition protocol. Images were acquired at 5 predefined zones with approximate coordinates with respect to the Fovea for the left (OS) and right (OD) eyes. The positive X-axis direction of the coordinate system points to the temporal side for both eyes.

AO imaging was performed using the Cellularis^®^ prototype 2.0 device, and for most patients, it was feasible to image both eyes. Each image taken by Cellularis^®^ corresponded to a field of view of 6.7° × 6.7° and recorded as 2048 × 2048 pixel. For the PR layer, we note that the optical resolution of the central foveal region corresponding to a circular area of radius 2.5° is rather poor. In order to systematically image the retina, five predefined regions of interest of the central macula were recorded. Figure 1 sketches the imaging protocol used for the acquisition of AO-TFI images.

The focal plane was adjusted based on the spherical equivalent refraction specified for each eye at the beginning of the image acquisition. Additionally, manual adjustments could be made via the software interface accompanying the acquisition device to ensure an optical focal plane. An internal yellow 580 nm LED target served as a fixation point for each of the five zones, while an external fixation target was provided for participants with reduced visual acuity and altered central fixation. We use this fixation target as an approximation to the location of the fovea, and later use it as the origin of our coordinate system for our measurements. However, this is only an approximation since the exact foveal location may differ slightly from the fixation target.

Finally, the resulting images from the confirmed healthy participants are gathered as a set we call “CEL01LUKS-normative”, consisting of 1029 RPE and 846 PR images.

**Figure 2:**
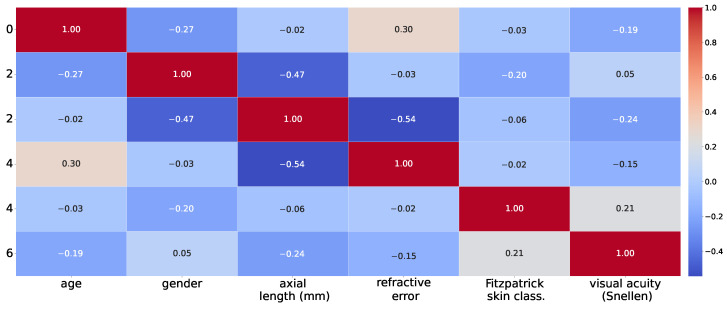
The Pearson correlation matrix of patient-attributes.

Figure 2 demonstrates the correlation of various patient attributes together.

### 2.2. Image Analysis and Quantification

We used the beta version RetiSense software package developed by EarlySight SA to analyze all RPE and PR images. The software is specifically designed for the analysis of AO images and uses Machine Learning and image processing to detect areas of interest, segment them and extract morphometric descriptors based on the segmented regions. This is described in Section 2.2.1.

We externally validated the performance of RetiSense cell detection by manual human-labeled counting of RPE cells as performed by five independent medical students and ensured that RetiSense outputs are aligned with manual counting values. This is described in Section 2.2.2.

For every RPE and PR image within the “CEL01LUKS-normative” set, the segmented regions provided by RetiSense are accumulated based on fixed-sized rectangular grids and the results are calculated for the entire database while filtering less reliable measurements. This is described further in Section 2.2.3.

#### 2.2.1. The RetiSense Software Package

RetiSense is specialized on the analysis of AO images and consists of four modes of operation: RPE cells, PR cells, hypo-reflective regions, and hyper-reflective regions. The reflective analyses are suitable for the quantification of retinal anomalies, such as drusen, and are hence useful for the characterization of retinal diseases, such as AMD. In this work, however, we use the RPE and PR cell analysis modes only to quantify healthy retinal cells and establish the normative database.

Internally, the software includes a module that identifies and filters out low-quality acquisitions—such as blurry, out-of-focus, or noisy regions of AO images—as well as retinal vessels that interfere with retinal cell segmentation.

For each analysis mode, the software automatically detects the relevant structures—RPE cell mosaics in RPE mode and circular Photoreceptor (PR) patterns in PR mode—within the unmasked regions of the image. Areas where the target structures are not clearly present are subsequently masked out, ensuring that the final analysis is restricted to regions containing the relevant cellular features that are clearly visible.

The software then detects all instances of the target structures within the remaining unmasked image regions. Two segmentation options are provided: the Voronoi tessellation that covers all the non-masked areas of the image and draws the boundaries of cells based entirely on the cell centroids, as well as the full segmentation mode that draws complete boundaries of the pigmented areas of RPE’s and the reflective circular contours around PR cells.

Since it is more established in the literature, we use only the Voronoi tessellation mode of segmentation in this study. As for the morphometric characterization of the detected cells, this mode computes the local density of cells, the area of the detected Voronoi regions (that correspond to the actual biological cells), their perimeter, as well as the number of neighbors that each cell has.

RetiSense (beta version v0.8.2-demo, as used in this study) processes a 2048×2048 pixel RPE or PR image in approximately 7 s on a standard consumer laptop, producing the corresponding segmentation output and morphometric characterizations.

**Figure 3:**
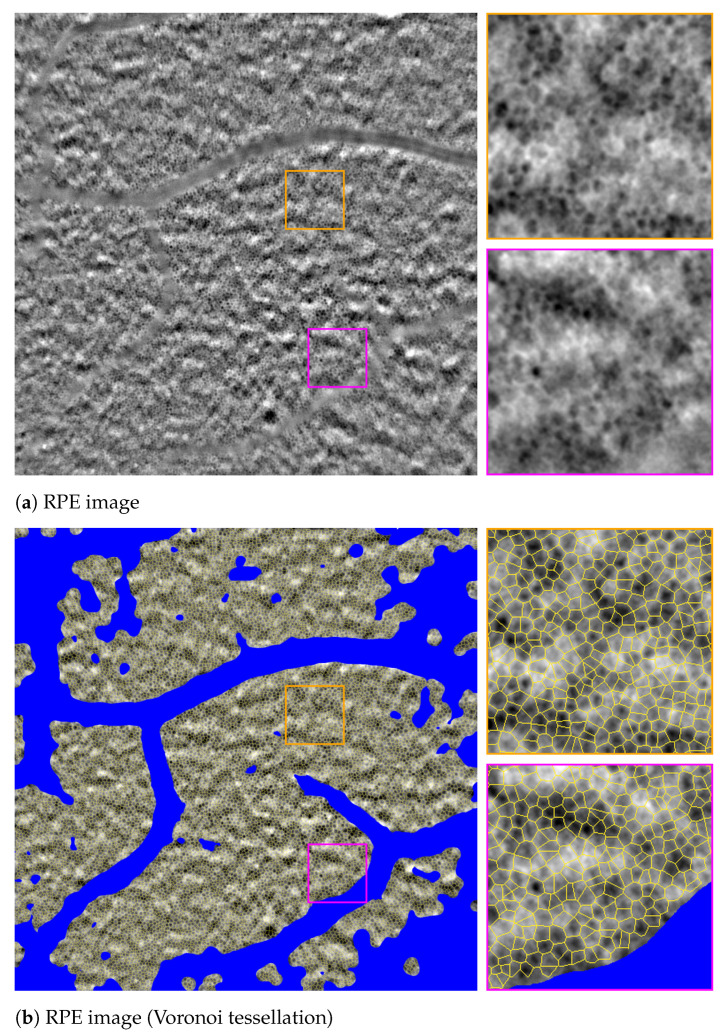

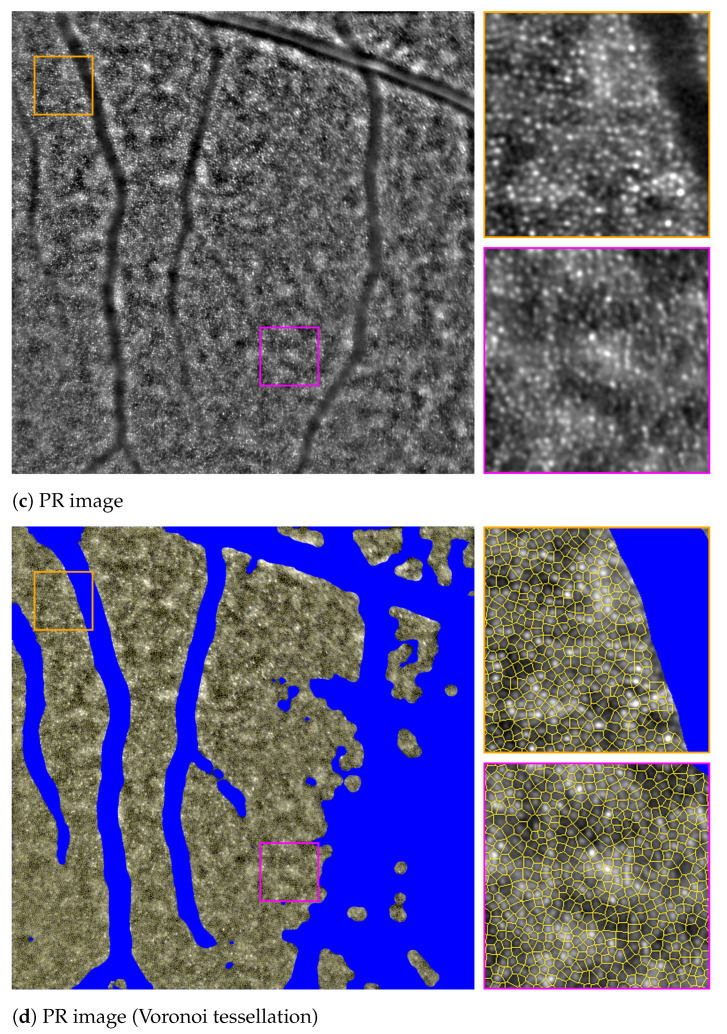
Examples of AO-TFI images captured by Cellularis^®^ and the segmentation (Voronoi tessellation) provided by RetiSense for RPE (**a**,**b**), and PR (**c**,**d**). The masked areas (in blue) are excluded from segmentation due either to low visibility, the presence of retinal vessels, or the absence of clearly visible RPE and PR cells, in RPE and PR analysis modes, respectively. Sample rectangular crops from images and their corresponding segmentation are magnified for visualization.

Figure 3 sketches Voronoi tessellations of two example RPE and PR images. The masked areas corresponding to both RPE and PR images appear as blue areas in the segmented images.

#### 2.2.2. Manual Validation of Cell Counting

To enable independent validation of RetiSense’s automated cell detection, a separate dataset of manual annotations—created entirely outside the scope of the present study—was used. An experienced retinal specialist with extensive expertise in AO-based cell counting trained five medical students to identify and mark RPE cell centroids. Each student independently annotated 90 image crops (300×300 pixels)), extracted from different RPE images of varying quality, using ImageJ2 Fiji [42]. These manual annotations served as an independent reference for evaluating the accuracy of the automated segmentation. The Intra Class Correlation (ICC3k), calculated according to the method described in [43] was 0.827 among the 5 raters and for the 90 patches (CI-95% in [0.76,0.88]), while the range between the minimum and maximum value of each counting normalized by the mean value of the 5 counts is calculated as 0.56 (±σ=0.198) on the average across the 90 patches, where σ denotes the standard deviation.

For all 90 image patches, the automatic RetiSense counts—adjusted for the proportion of unmasked image area—fell within the range of the minimum and maximum manual counts, demonstrating consistency with the annotations of trained human raters.

**Figure 4:**
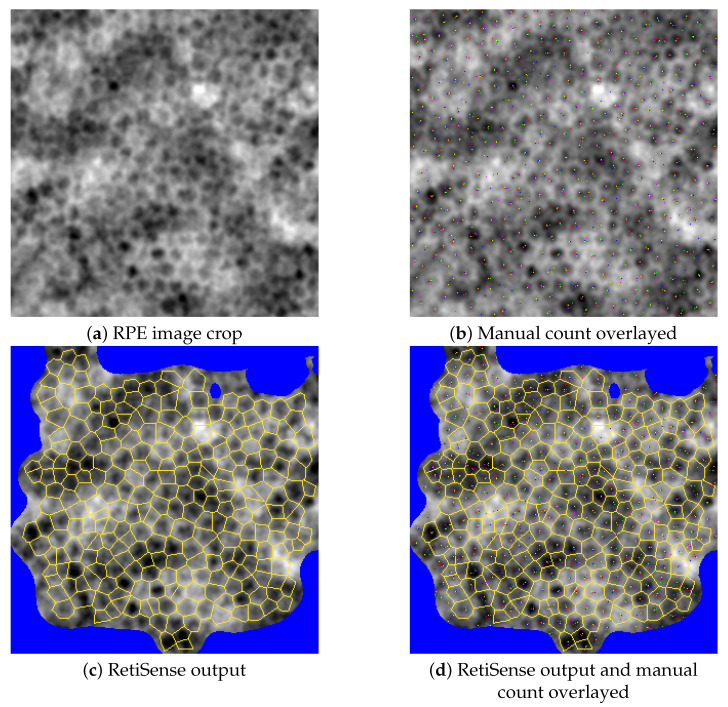
(**a**) A sample crop of a 300×300 RPE image, (**b**) five independent manual cell counts overlaid on the raw image where each color represents an individual’s annotations, (**c**) automatic counting output from RetiSense, and (**d**) manual counts overlaid on the RetiSense output.

Figure 4 presents a sample RPE image patch alongside the manual annotations from the trained raters and the RetiSense output. While the manual annotations vary slightly in the exact placement of cell centroids, there is general agreement on cell detection. The automated RetiSense counts closely align with the majority of the manual annotations. It should be noted that these manual labels were not used to train the Machine Learning algorithms used within RetiSense, as this was a completely independent procedure.

It should also be noted that, unlike the systematic validation procedure used for RPE cell detection, the automated PR detection pipeline in RetiSense was only assessed through visual inspection of multiple samples, without a comparable quantitative validation

In contrast to RPE cells, Photoreceptor (PR) cells are typically more clearly visible, making their detection more straightforward. Due to this consistently high visibility and lower structural variability, visual inspection was deemed sufficient to confirm the reliability of the automatic PR cell detection.

#### 2.2.3. Analysis of the “CEL01LUKS-Normative” Set

We ran the RetiSense software (beta version v0.8.2-demo) on all 1029 RPE and 846 PR images of the “CEL01LUKS-normative” set described at Section 2.1. For each image, the software requires to specify the pixel size in μm, which we calculate based on the axial length of the corresponding eye and according to the Bennett-Littmann formula [44] of Equation (1):

(1) $$\mathrm{pixel}\;\mathrm{size}\left(\mu m\right)=0.504\times 0.01306\left(\mathrm{axial}\;\mathrm{length}(\mathrm{mm})-1.82\right)\times 6.5.$$

Images with more than 90% of their area masked, as determined by RetiSense, were excluded due to insufficient visibility of RPE or PR structures. We call this subset of the study the “CEL01LUKS-normative-mosaic” set.

As described earlier in Section 2.1.3, AO images are acquired at 5 pre-defined zones for each eye as shown in Figure 1. After extracting the segmentation data from RetiSense—including each detected cell along with its morphometric properties and pixel coordinates relative to the image—we divided each 2048×2048 image into 64 grid squares of 256×256 pixels and identified all cells located within each square.

For each cell, its position within a grid square is approximated using the coordinate of its center pixel. This local pixel coordinate is then converted to a global macular coordinate in degrees relative to the fovea, as illustrated in Figure 1, assuming a fixed image field of 6.7° × 6.7°. The conversion requires the global coordinate of the image center relative to the fovea. Thi is estimated by the Cellularis^®^ device based on the participant’s fixation on the predefined fixation target during image acquisition (fixation-based coordinate system). As an additional filtering stage, only grid squares of 256 × 256 containing at least 200 cells were kept for further analysis. This is to exclude detected cells that constitute small islands within masked areas whose measurements may not be accurate.

We define the eccentricity in degree with respect to the Fovea as in Equation (2):

(2) $$e^{\circ }=\mathrm{sign}\left(x^{\circ }\right)\times \sqrt{{\left(x^{\circ }\right)}^{2}+{\left(y^{\circ }\right)}^{2}},$$

where the sign(·) function specifies the sign of the x-value of the calculated coordinate according to the global coordinate system for each eye, and hence the temporal and nasal sides will be assigned as positive and negative eccentricities, respectively.

As the last filtering step and only for PR images, we omit PR cells detected within eccentricities between −2.5° and 2.5°. This is due to optical properties of the device, whose PR measurements around the Fovea may be inaccurate. As a result of the filtering stages described above, we obtained the “CEL01LUKS-normative-mosaic (filtered)” subset. To assess whether the filtering introduced any demographic bias, its characteristics were compared to those of the original parent datasets, as shown in Table 1. For simplicity, we do not stratify age based on decades and we suffice with the mean and standard deviation for each subset.

Once the global coordinate of all the detected cells are acquired, we quantize every cell coordinate to 25 equal-sized bins across all eccentricities. We then average the morphometric descriptors derived from RetiSense within every bin. We next present the results in Section 3.

## 3. Results

Table 2 and Table 3 summarize the quantitative descriptors extracted across the whole “CEL01LUKS-normative-mosaic (filtered)” set as detailed earlier in Section 2 and for RPE and PR images, respectively. The results are stratified based on gender, and summaries are calculated based on mean, median, standard deviation, 25% percentile, as well as 75% percentiles for all the morphometric features, i.e., local density (mm^−2^), number of neighbors of the Voronoi tessellations, area (μm^2^) and perimeter (μm).

### 3.1. Eccentricity Profile

**Figure 5:**
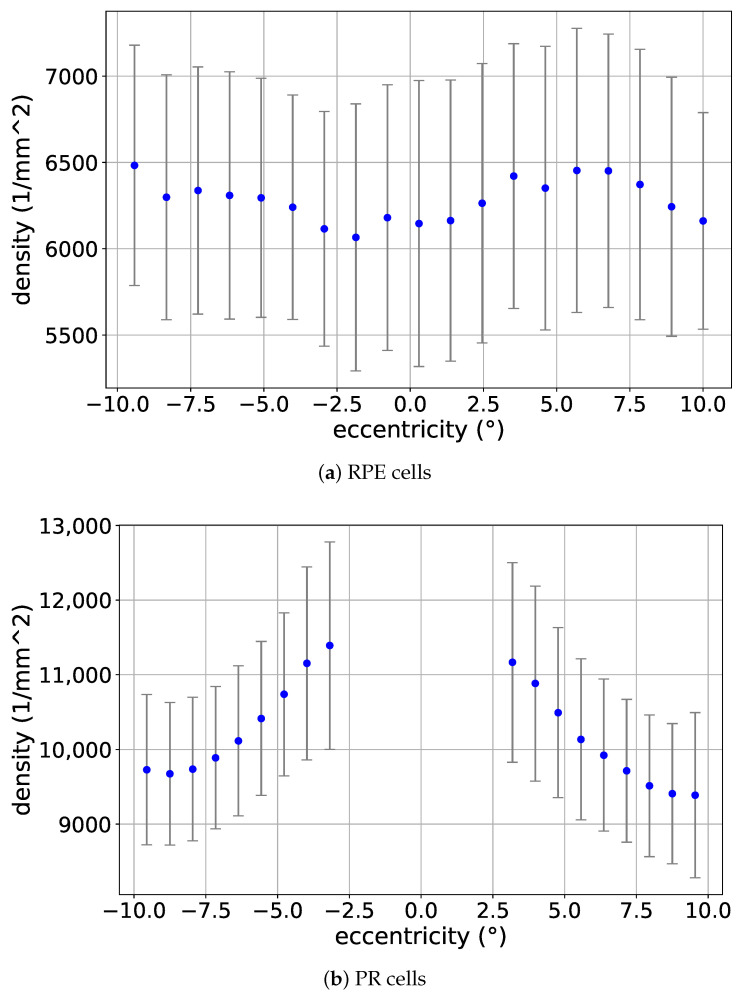
Retinal cell densities from the “CEL01LUKS-normative-mosaic (filtered)” set as a function of eccentricity from the Fovea. Positive and negative eccentricities correspond to the temporal and nasal sides, respectively. The error bar around each averaged data point has a width of two standard deviations. The eccentricity 0° corresponds to a fixation target and only approximatively corresponds to the Fovea.

To examine the variation of cell density across different regions of the Macula, and as was detailed earlier in Section 2.2.3, Figure 5a,b sketch the profile of the local density of RPE and PR cells with respect to the eccentricity from the Fovea, respectively.

**Figure 6:**
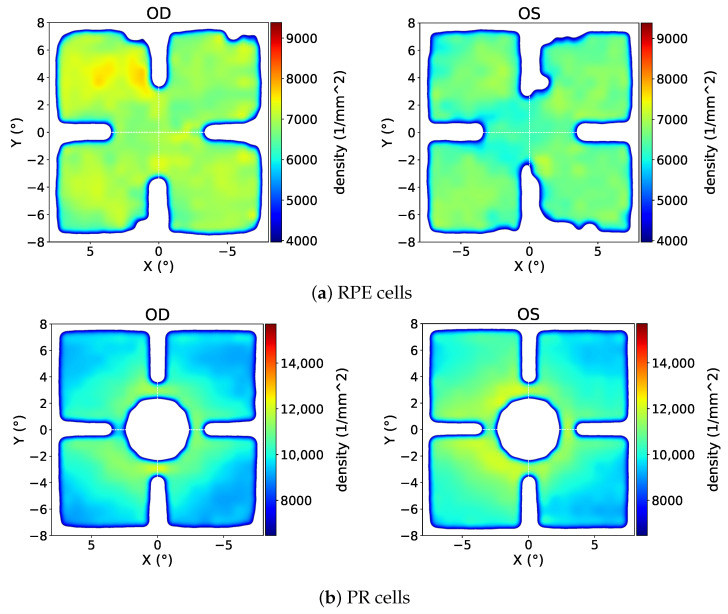
Spatial distribution of retinal cell densities from the “CEL01LUKS-normative-mosaic (filtered)” set as heatmaps for both eye sides. A Gaussian smoothing with σ≈ the sampling distance was applied. Areas with lack of samples are transparent or white.

In order to see the spatial profile of the density with more details and for both eye sides, heatmaps of density are presented in Figure 6.

As observed from the figures, while the distribution of RPE cells with respect to the eccentricity does not show a clear pattern, the PR cell density decreases with increasing eccentricity. This RPE finding somewhat differs from established literature showing foveal density peaks, likely due to our coordinate system limitations.

**Figure A1:**
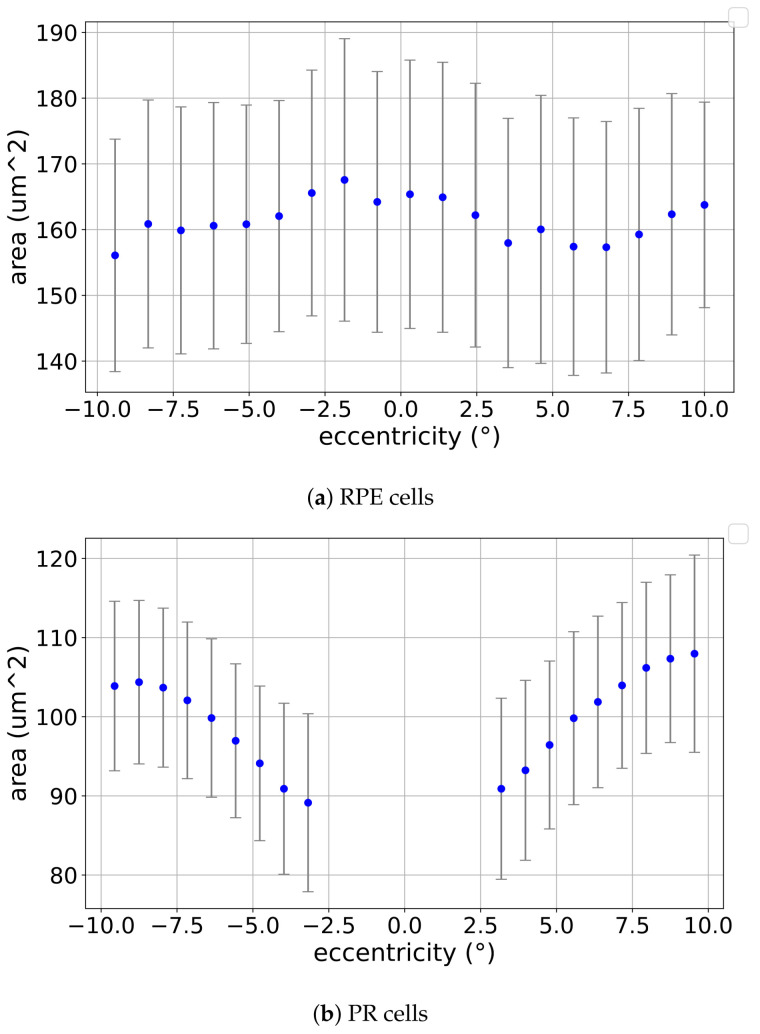
Area of cells as a function of eccentricity from the fovea for (**a**) RPE cells and (**b**) PR cells.

**Figure A2:**
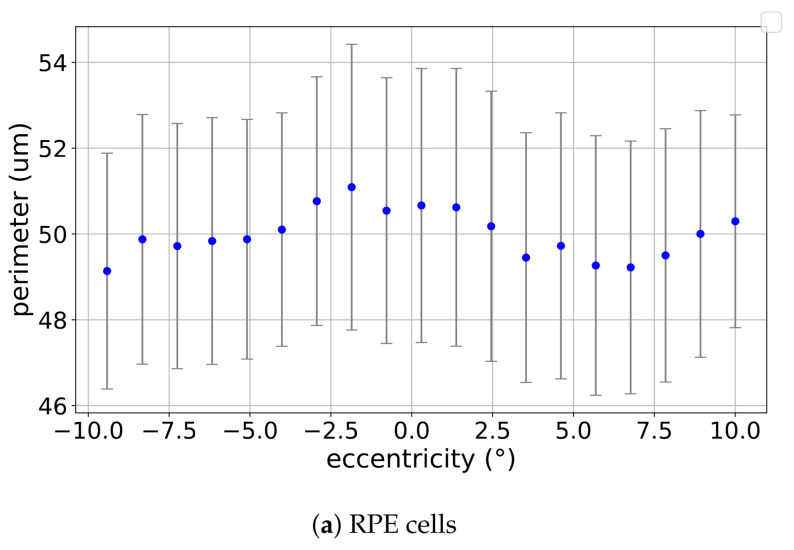

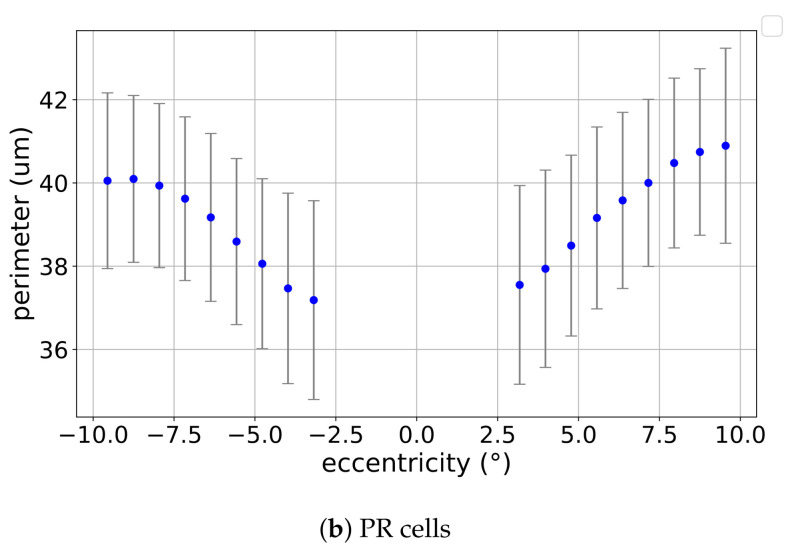
Perimeter of cells as a function of eccentricity from the fovea for (**a**) RPE cells and (**b**) PR cells.

**Figure A3:**
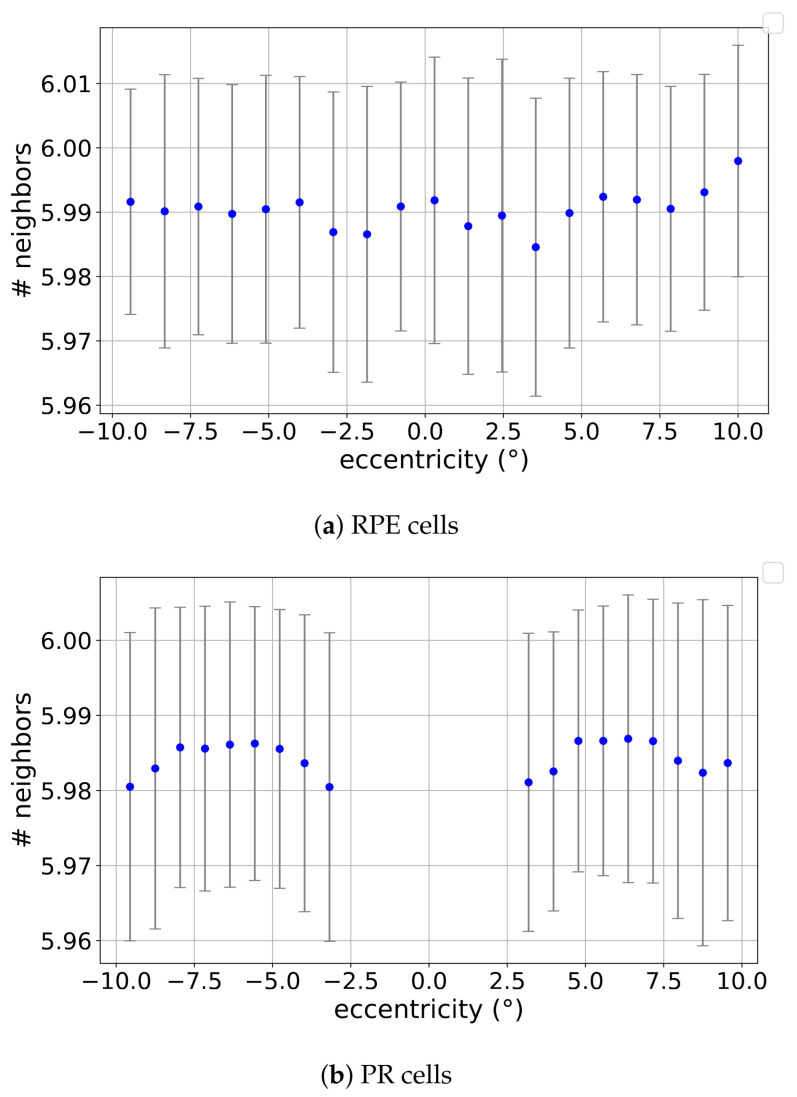
Number of neighbors in Voronoi tessellation of detected cells as a function of eccentricity from the fovea, for (**a**) RPE cells and (**b**) PR cells.

**Figure A4:**
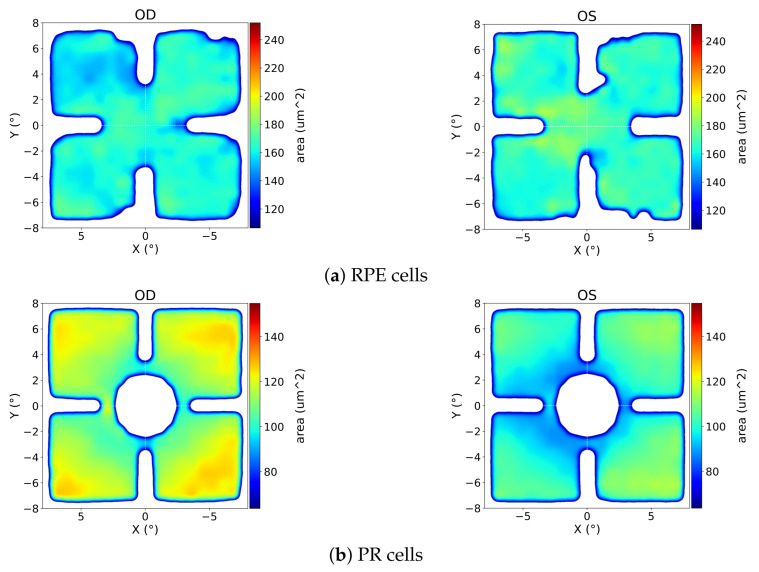
Spatial distribution of retinal cell areas from the “CEL01LUKS-normative-mosaic (filtered)” set as heatmaps for both eye sides. A Gaussian smoothing with σ≈ the sampling distance was applied. Areas with insufficient sampling appear transparent or white.

**Figure A6:**
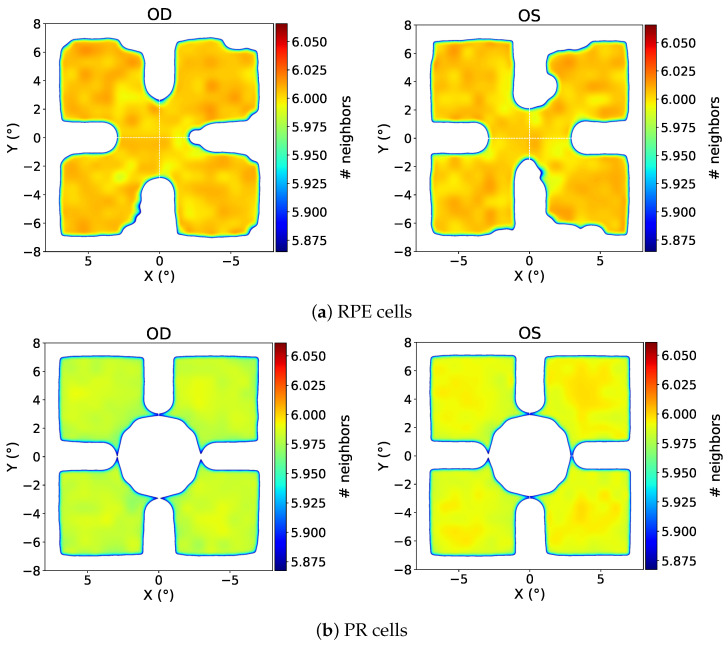
Spatial distribution of the number of neighbors of retinal cells based on Voronoi tessellations from the “CEL01LUKS-normative-mosaic (filtered)” set, shown as heatmaps for both eye sides. A Gaussian smoothing with σ≈ the sampling distance was applied. Areas with insufficient samples appear transparent or white.

In the supplementary Appendix A, we present the results of other morphometric descriptors, i.e., area, perimeter and the number of neighbors of the Voronoi tessellation cells with respect to eccentricity as supplementary Figure A1, Figure A2 and Figure A3, and as spatial heatmaps in Figure A4, Figure A4 and Figure A6, respectively.

AO-TFI imaging in this study is optimized for high-reflectance cone photoreceptors and the overlying RPE mosaic; rod outer segments and deeper retinal layers lie below the system’s resolution and reflectance threshold. Thus, all reported PR metrics (density, morphology, spatial arrangement) refer specifically to cone populations and should not be interpreted as total photoreceptor counts.

In order to compare the morphometric descriptors of PR and RPE cells for every given eye, we compute the ratio of the descriptor values at every similar coordinate of the same eye for all acquisitions. Following the procedure discussed earlier in Section 2.2.3, for both RPE and PR acquisitions, after having aggregated every detected cell to the centroid of its corresponding rectangular grid of size 256×256 and after filtering out the rectangles with very small number of cells detected, we find the corresponding PR rectangles for a given RPE rectangle based on their centroid coordinates. So for a given eye for which both acquisition types are available, if a PR rectangle has very close centroid coordinates to a given RPE rectangle, we associate them together and compute the ratio of its morphometric descriptors. Otherwise, if the coordinate-based matching fails for a rectangle, we do not take it into account for the ratio-based analysis. The matched rectangles are then binned based on their eccentricities.

**Figure 7:**
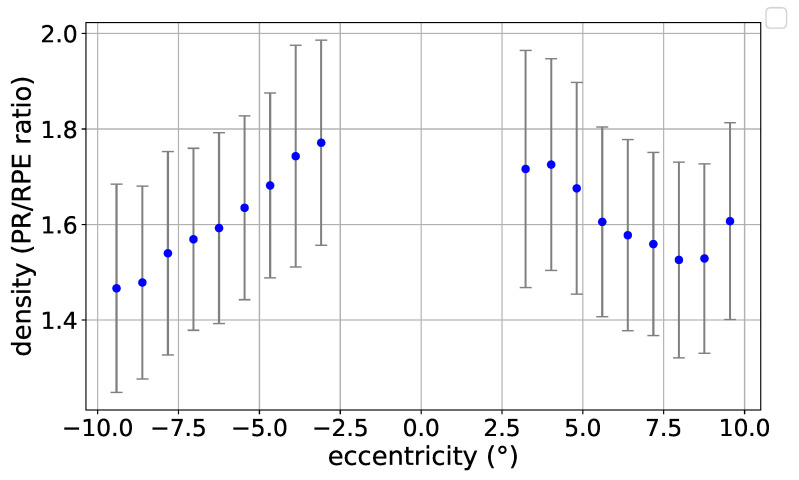
The ratio of PR over RPE for cell density as a function of eccentricity from the Fovea.

**Figure A7:**
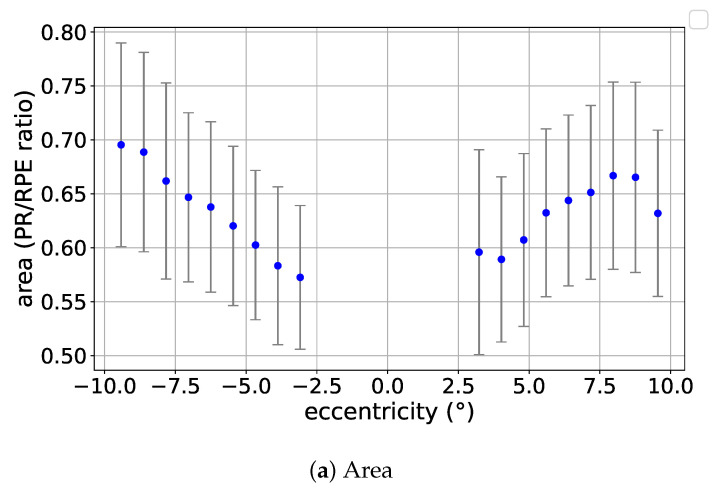

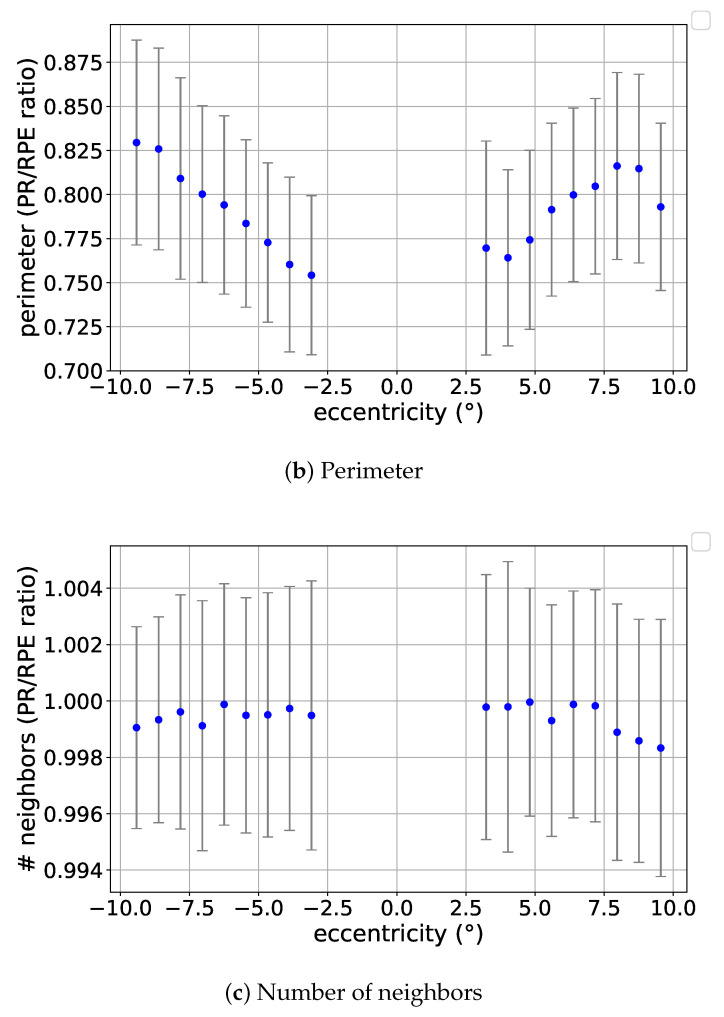
The ratio of PR over RPE values as a function of eccentricity from the fovea for (**a**) cell area, (**b**) cell perimeter, and (**c**) the number of neighbors in Voronoi tessellation cells.

Figure 7 shows this ratio profile for cell density as a function of eccentricity. Likewise, the ratio profile for other morphometric descriptors are presented as the supplementary Figure A7.

Previous histological and adaptive optics studies have shown that the PR/RPE ratio exhibits a biphasic profile, with a central peak reflecting foveal cone specialization and a second inflection in the parafoveal region [45,46]. Our current analysis does not capture this pattern, as our fixation-based coordinate system and exclusion of the central ±2.5° region limit resolution of these critical zones. Future studies should integrate precise anatomical co-registration and high-resolution central imaging to reveal the expected biphasic PR/RPE ratio.

### 3.2. Age Profile

**Figure 8:**
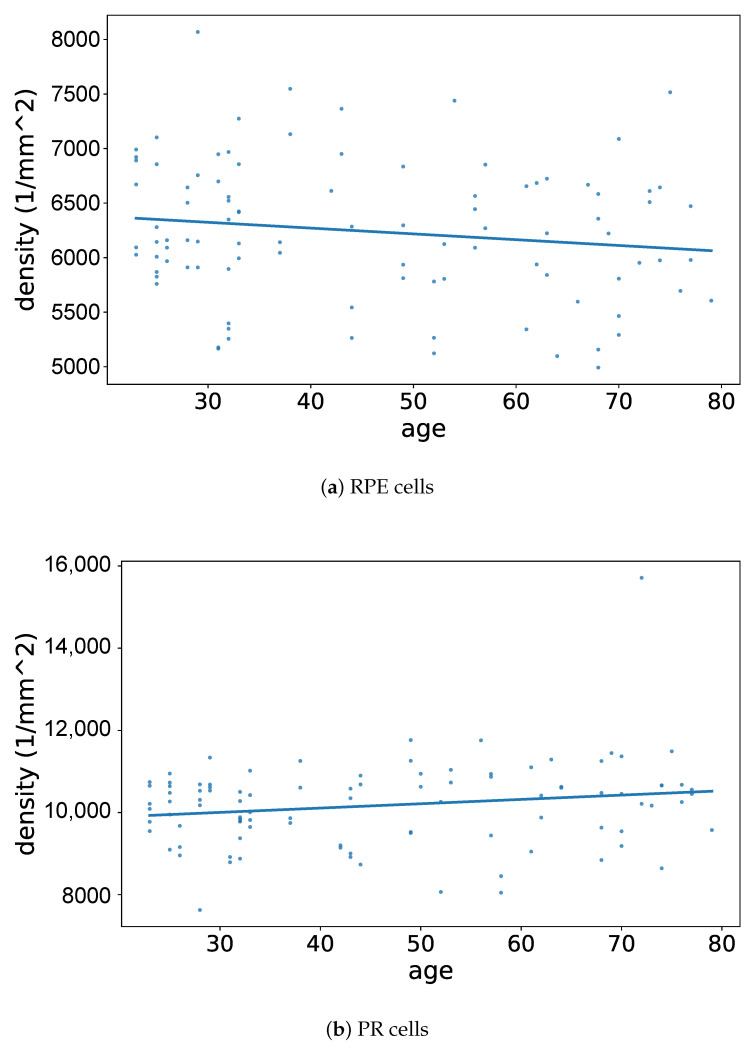
Cell density as a function of age. Cell density is defined as the total number of identified cells per visible (non-masked) area.

To see the variation of cell characteristics with respect to age, Figure 8a and Figure 8b sketch RPE and PR cell densities as a function of the age of the participants, respectively.

While the RPE cell density seems to have a slight decline with age, the PR cell density even suggests a slight increase, likely due to statistically insignificant variations in the data. Moreover, our parafoveal imaging region contains a high rod proportion, and age-related decreases in rod reflectance may reduce detection efficiency, masking true rod loss and producing a flat PR density curve. Future work should employ reflectance-calibrated imaging or subtype-specific segmentation to accurately capture rod and cone density changes with age.

Only healthy participants were included in this study, which may help explain why no age-related decline in PR cell density was observed. As visual acuity generally remains stable in healthy individuals even at advanced age, it is reasonable to assume that Photoreceptor counts do not significantly decrease. In contrast, age-related changes in RPE cell density are less well understood at the cellular level in vivo. The moderate decline observed in our data may partly reflect methodological limitations, particularly the exclusion of poorly visible or structurally irregular regions from analysis.

As described earlier, an important limitation of our PR quantification pipeline concerns the validation of Photoreceptor segmentation, which was not validated independently, e.g., similarly to our RPE identification pipeline that was manually validated by trained medical students (ICC = 0.827). Future studies should implement comprehensive quantitative validation for all measured cell types to ensure methodological consistency and reliability.

The RetiSense software is designed to detect regular cellular patterns—specifically, the honeycomb-like arrangement of RPE cells and the circular reflective patterns of PR cells. Regions with highly irregular or pathological morphology are filtered out to ensure robust cell-based measurements. Such regions are better suited for analysis using the software’s hypo- and hyper-reflective modes, which are more appropriate for identifying disease-related patterns, including geographic atrophies or severely deformed cells. A detailed analysis of these pathological features will be addressed in future work, where we focus on comparing healthy and diseased retinal tissue.

However, as an alternative to cell density, to better see the age-related decline of cell structures, we introduce another measure, the “visible cell mosaic area ratio”.

This equals the ratio of the area of visible cells over the total visible areas across the image. Note that the denominator is measuring only the visible and in-focus parts of the image, and excludes the areas on which we are not confident about the underlying structure. This is to minimize the effect of the imaging artifacts, and the fact that, as compared to the younger participants, it is typically more difficult to image older participants with high image qualities. So the images of older eyes would typically contain more areas with lower visibilities, on which we are not sure about whether the underlying imaged area belongs to healthy cells, or other structures such as atrophies. By measuring the ratio of visible cells over the entire visible areas, therefore, we are excluding this bias and ensuring a meaningful criteria that focuses on the underlying biological factors, rather than imaging artefacts.

**Figure 9:**
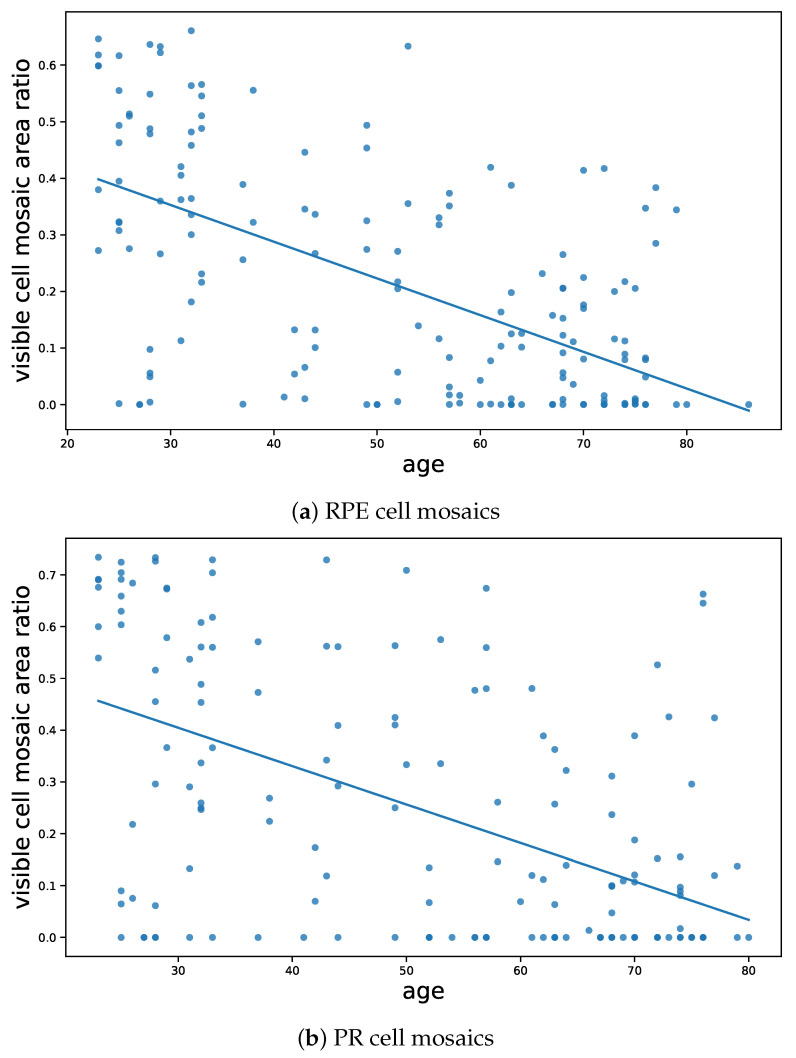
The ratio of visible cell mosaic areas over non-mosaic visible areas for (**a**) RPE cells, and (**b**) PR cells.

Figure 9 shows this ratio for both RPE and PR cells. As is obvious from the figure, the ratio of visible cell mosaic areas over all visible areas has a sharp decline with respect to age and for both RPE and PR cells.

### 3.3. Correlation with Independent Variables

**Figure 10:**
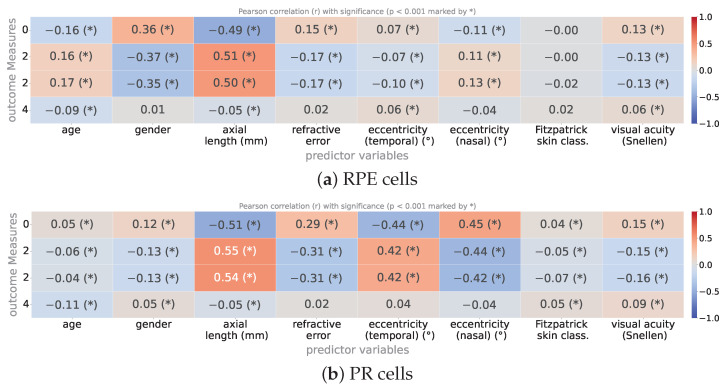
Pearson correlation matrix showing the linear association between outcome morphometric descriptors and individual metadata predictors for (**a**) RPE cells and (**b**) PR cells. The values represent Pearson correlation coefficients (*r*), ranging from −1 to 1, indicating the direction and strength of the association. Statistical significance of each correlation was evaluated under the null hypothesis of zero correlation (r=0), and an asterisk next to the correlation value “r(∗)” denotes *p*-value < 0.001.

To see the effect of patient-related variations other than age, here we study the effect of other independent variables such as eye’s axial length or refractive error on the outcome measurements, i.e., the morphometric descriptors of cells. We calculate based on each one of the independent variables alone, to which extent the variations in the measured descriptor are predictable when a simple linear regression is used. Equivalently, we calculate the Pearson correlation coefficients of all measurements with every one of the independent variables. The resultant *r* correlation matrix is shown in Figure 10a and Figure 10b, for RPE and PR images, respectively.

For most of the measured descriptors, there does exist statistically significant dependence on most of the independently-considered variables. Most notably, we see clear correlation of the outcome measures, i.e., density, area and perimeter with axial length of the eyes and for both RPE and PR images. So the longer the eyes, the larger the cell areas, as our study confirms.

## 4. Discussion

This study aimed at establishing a normative database characterizing healthy human retinal cells, i.e., Retinal Pigment Epithelium cells (RPE’s) and cone Photoreceptors (PR’s) using in-vivo imaging. This included 171 eyes from 104 healthy participants of various age groups (23 to 80 years old), making it the largest-scale study ever reporting cellular-level characterizations of the retina.

The study was carried out at the Luzerner Kantonsspital (LUKS), Switzerland, where ophthalmologists selected healthy eyes from a larger pool of participants, following comprehensive checkups based on SD-OCT and Fundus imaging.

We used the custom RetiSense software package (beta version v0.8.2-demo) that comes with the imaging device to automatically segment retinal cells from Adaptive Optics based imaging. The software automatically masks low-quality or non-visible regions of the images, including retinal vessels. The remaining unmasked areas are analyzed for the presence of either RPE, or PR mosaics. For each mode, cell centers are detected and used to provide Voronoi tessellations. The segmented cells were characterized with morphometric descriptors such as cell density and area, providing detailed and granular normative statistics stratified by various independent parameters such as retinal eccentricity, age, etc.

The automated RPE cell counts were compared to manual annotations performed independently by five trained medical students not involved in this study. The software consistently produced values within the range defined by the minimum and maximum of the manual counts.

The images were acquired using Cellularis^®^ prototype 2.0 device, developed by EarlySight SA, Geneva, Switzerland. Each eye was imaged at up to 6 different coordinates with respect to the fovea, where each image covers an area of approximately 6.7° × 6.7°. Both RPE mode and PR mode images were acquired for most of the eyes and at equal or similar coordinates.

The Cellularis^®^ device uses the AO-TFI imaging technology that offers non-invasive, cellular-resolution imaging of the retina and is increasingly being adopted in clinical practice. AO imaging has transitioned from research to clinical practice, with established applications in inherited retinal diseases and Age-related Macular Degeneration (AMD). The technology is now routinely integrated into clinical trials as a sensitive outcome measure and has achieved regulatory approval in several countries [47,48]. A crucial step in the widespread adoption of cellular-level imaging in clinical practice is to establish reliable and large-scale normative datasets. This study, with its relatively large number of participants aims at providing such a foundation.

This normative database, with its rich number of participants of various ages, and the eccentricities of up to 10°, when complemented by future studies on retinal anomalies, will ultimately pave the way for identifying robust cellular biomarkers for the early detection, progression tracking, and treatment monitoring of retinal diseases. Exclusion of the central ±2.5° foveal region from our Photoreceptor analysis, necessitated by technical constraints, however, omits the retinal area with the highest cone density and most pronounced inter-individual variability, as established in histology studies e.g., by [45], potentially leading to underestimation of absolute peak values and variation.

### 4.1. Results in the Context of the Literature

Our observation of minimal PR density change with age contrasts with histological evidence. Curcio and colleagues demonstrated a 30% rod loss in central retina over adulthood, alongside stable foveal cone counts and clear RPE density gradients with both eccentricity and age [46,49]. The apparent discrepancy likely stems from methodological differences: in-vivo flood-illumination imaging lacks the resolution to resolve tightly packed foveal cells, and from coordinate registration errors that blur steep density gradients over small spatial scales.

Our finding of flat RPE density across 0° to 9° eccentricity contrasts with well-established literature demonstrating foveal density peaks. Multiple histological studies have shown RPE density peaks at the foveal center: Panda-Jonas et al. reported foveal RPE density of 4220 ± 727 cells/mm² decreasing to 3002 ± 460 cells/mm² peripherally [50], while Liu et al. found a negative slope of −123 cells/mm²/degree from a foveal peak of 6913 cells/mm² [40]. Recent in-vivo studies using higher-resolution adaptive optics have confirmed these patterns, with Baraas et al. measuring foveal RPE densities ranging from 5621–9677 cells/mm² [51].

Several methodological factors likely explain this discrepancy. Firstly, our fixation-based coordinate system introduces positional uncertainty that may blur steep density gradients occurring over small spatial scales. Secondly, the established foveal RPE density peak corresponds to smaller, more densely packed cells that may challenge our current resolution and automated detection capabilities. Finally, all established studies used either ex-vivo histological analysis or higher-resolution adaptive optics with precise anatomical registration, methodologies that exceed the precision of our in-vivo flood-illumination approach.

This discrepancy highlights fundamental limitations of our current methodology and reinforces that our findings should be interpreted as preliminary technical validation rather than definitive anatomical characterization. Future work must prioritize anatomical co-registration and improved central retinal resolution to accurately capture established RPE topographical patterns.

### 4.2. Technological Limitations

Several important limitations of the present study must be acknowledged when interpreting our findings and planning future work.

Our analysis relies on a fixation-based coordinate system, in which retinal eccentricities are estimated from the patient’s gaze on an internal fixation target. Unlike anatomically registered coordinates aligned to the foveal pit or optic disc margin, this approach introduces systematic positional uncertainty. Prior adaptive optics studies have demonstrated offsets of up to several arcminutes between fixation centers and anatomical foveal specializations, driven by both fixation instability and interindividual variability [52,53]. Consequently, our eccentricity estimates may be imprecise, particularly near the foveal center where cone density gradients are steepest.

We excluded Photoreceptor data within ±2.5° of fixation due to the optical resolution limits of the AO-TFI system in the central retina. This region corresponds to the foveal cone mosaic’s peak density (>150,000 cones/mm², foveal cone bouquet) and underlies the highest visual acuity in humans [45]. By omitting this critical zone, our data cannot capture the full topographical range of Photoreceptor variation, and comparisons with normative ex-vivo histological databases such as [45] are fundamentally constrained.

To ensure robust automated cell detection, regions of poor image quality or irregular cellular patterns were excluded. Although necessary for measurement reliability, this selection may bias our sample toward areas of optimal reflectance and morphology, particularly in older subjects. Such bias could artificially attenuate age-related trends in cell density.

Without precise anatomical registration, our measurements cannot be directly mapped to clinical coordinate systems (e.g., ETDRS grids), nor reliably compared across imaging modalities. This limits immediate clinical applicability, as diagnostic thresholds and progression metrics for diseases such as AMD and inherited Photoreceptor dystrophies rely on anatomically anchored measurements of foveal and parafoveal regions.

Another potential limitation of our approach is that magnification scaling was performed using the Bennett-Littmann formula based solely on axial length, while recent adaptive optics studies have shown that empirical validation and additional biometric parameters may improve accuracy [54,55].

Addressing these limitations will be crucial for translating AO-TFI imaging to clinical practice: Firstly, integrating simultaneous OCT volumes or fundus landmarks will enable accurate anatomical co-registration. Automated detection of the foveal pit on OCT and registration with flood-illumination montages can reduce positional uncertainty. Secondly, hardware improvements to enhance axial and lateral resolution will allow direct visualization of foveal cones and finer characterization of the RPE mosaic. Finally, validating in-vivo density measurements against ex-vivo histology in donor eyes will help calibrate our imaging metrics and quantify systematic biases.

## 5. Conclusions

In this study, we presented a large-scale, automated characterization of healthy human retinal cells, i.e., RPE and PR cells using AO-TFI imaging technology. Through analysis of 171 eyes from 104 healthy participants, we derived a normative morphometric data across a wide age range and at multiple retinal eccentricities.

Our results showed that PR cell density decreases with increasing eccentricity, while RPE cell density does not exhibit a consistent spatial trend but instead declines linearly with age. These patterns are consistent with existing literature and provide in vivo confirmation at an unprecedented scale and resolution. The average RPE and PR cell densities were quantified as approximately 6313 cells/mm^2^ and 10,207 cells/mm^2^, respectively, with automated RPE measurements validated against manual annotations.

The fully automated and user-independent nature of our pipeline ensures reproducibility and scalability, making it well-suited for clinical environments. The normative database established here strengthens the foundation for the clinical application of AO-TFI imaging and enables objective comparisons in pathological conditions.

Ultimately, this normative dataset, when paired with future studies focusing on retinal anomalies, will support the identification of reliable, quantitative biomarkers advancing the early diagnosis, monitoring, and personalized management of retinal diseases.

## Figures and Tables

**Table 1:** Demographics summary of the different subsets of this study. “CEL01LUKS-normative” consists of all the confirmed healthy cases of the study (Section 2.1.3. The “CEL01LUKS-normative-mosaic” is a subset with at least 10% of cell areas (Section 2.2.3), while the “CEL01LUKS-normative-mosaic (filtered)” follows filtering stages described in Section 2.2.3.

	Female						Male						Total					
	**Participants**	**Eyes**	**RPE**	**PR**	**Age** **(Mean)**	**Age** **(Std)**	**Participants**	**Eyes**	**RPE**	**PR**	**Age** **(Mean)**	**Age** **(Std)**	**Participants**	**Eyes**	**RPE**	**PR**	**Age** **(Mean)**	**Age** **(Std)**
CEL01LUKS-normative	58	100	582	492	51.8	19.1	46	72	447	354	58.5	17.2	104	172	1029	846	54.8	18.5
CEL01LUKS-normative-mosaic	45	76	296	217	47.2	17.67	37	51	204	148	58.7	16.97	82	127	500	365	52.39	18.19
CEL01LUKS-normative-mosaic (filtered)	42	71	222	217	45.79	17.32	33	47	121	148	58.55	16.73	75	118	343	365	51.40	18.11

**Table 2:** RPE image statistics summary of the “CEL01LUKS-normative-mosaic (filtered)” set.

	Female					Male					Total				
	**Mean**	**Median**	**Std**	**25%**	**75%**	**Mean**	**Median**	**Std**	**25%**	**75%**	**Mean**	**Median**	**Std**	**25%**	**75%**
local density (mm^−2^)	6488.09	6422.58	741.80	5995.38	6947.01	5886.64	5888.19	611.53	5447.63	6281.45	6313.56	6257.57	757.38	5823.85	6765.04
number of neighbors	5.99	5.99	0.02	5.98	6.00	5.99	5.99	0.02	5.98	6.00	5.99	5.99	0.02	5.98	6.00
area (μm^2^)	156.14	155.70	17.90	143.95	166.80	171.72	169.83	17.99	159.20	183.57	160.67	159.81	19.27	147.82	171.71
perimeter (μm)	49.18	49.19	2.83	47.30	50.88	51.46	51.22	2.71	49.56	53.37	49.85	49.77	2.98	47.92	51.62

**Table 3:** PR image statistics summary of the “CEL01LUKS-normative-mosaic (filtered)” set.

	Female					Male					Total				
	**Mean**	**Median**	**Std**	**25%**	**75%**	**Mean**	**Median**	**Std**	**25%**	**75%**	**Mean**	**Median**	**Std**	**25%**	**75%**
local density (mm^−2^)	10,323.61	10,261.17	1214.41	9496.71	11,033.76	10,024.69	9919.62	1340.54	9077.56	10,836.12	10,207.21	10,151.19	1273.39	9332.74	10,969.40
number of neighbors	5.99	5.99	0.02	5.98	6.00	5.98	5.99	0.02	5.97	6.00	5.99	5.99	0.02	5.98	6.00
area (μm^2^)	98.20	97.45	11.54	90.63	105.30	101.53	100.81	13.69	92.28	110.16	99.50	98.51	12.53	91.16	107.15
perimeter (μm)	38.89	38.77	2.33	37.37	40.35	39.56	39.46	2.66	37.78	41.29	39.15	39.02	2.49	37.52	40.68

**Figure A5:**
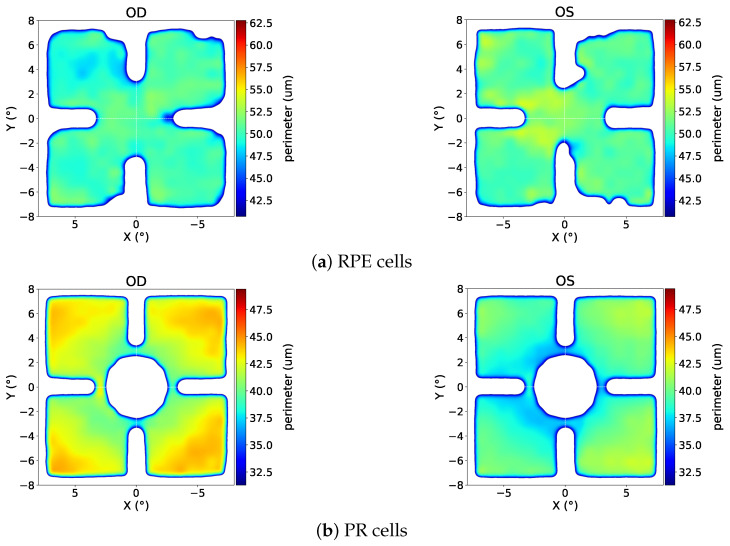
Spatial distribution of retinal cell perimeters from the “CEL01LUKS-normative-mosaic (filtered)” set as heatmaps for both eye sides. A Gaussian smoothing with σ≈ the sampling distance was applied. Areas with insufficient samples appear transparent or white.

**Figure A8:**
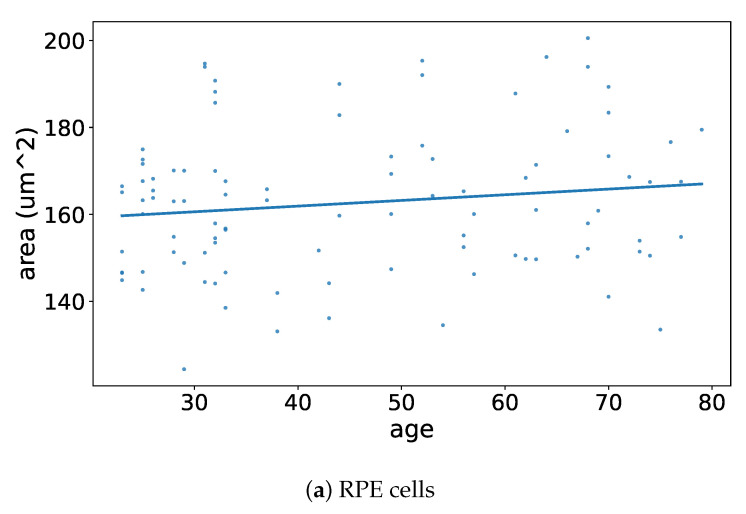

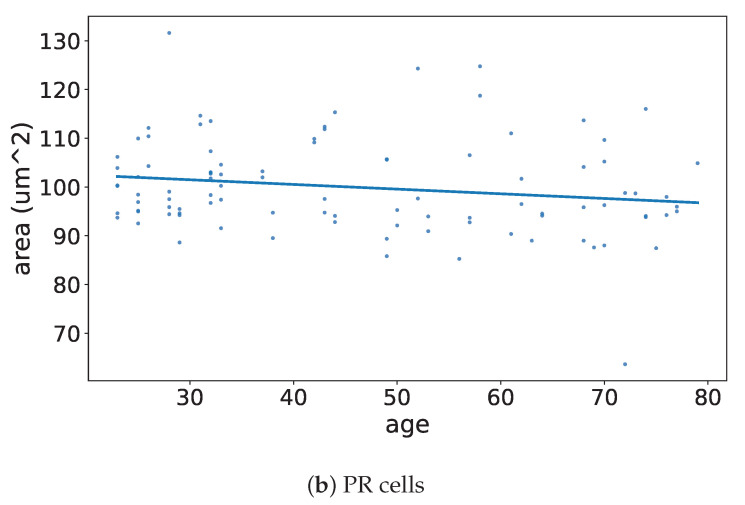
Area of cells as a function of age for (**a**) RPE cells and (**b**) PR cells.

**Figure A9:**
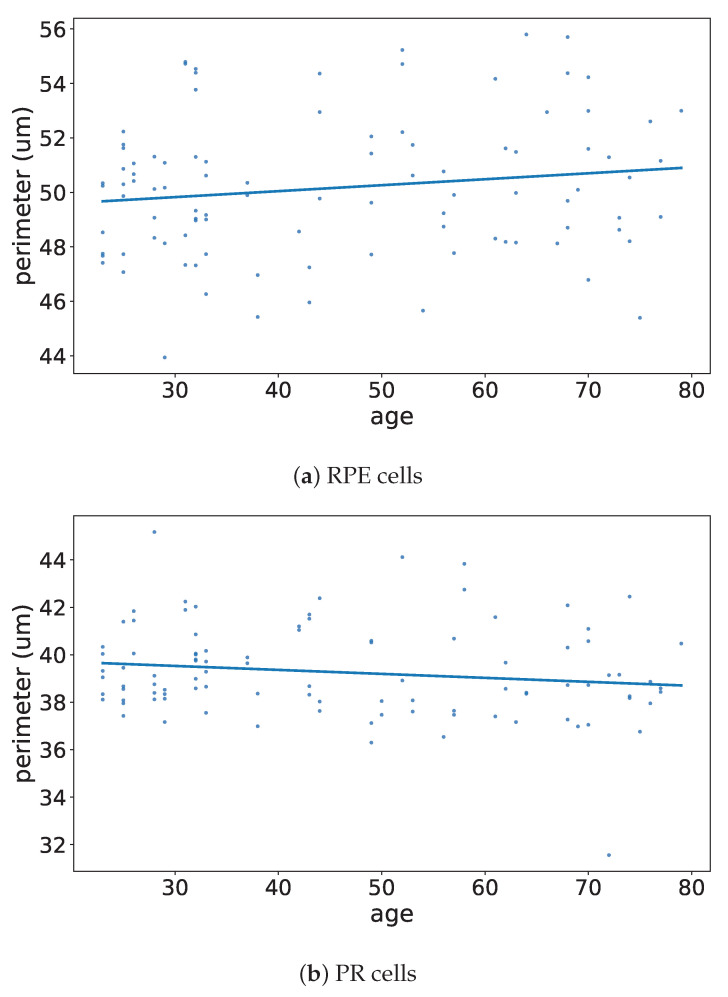
Perimeter of cells as a function of age for (**a**) RPE cells and (**b**) PR cells.

**Figure A10:**
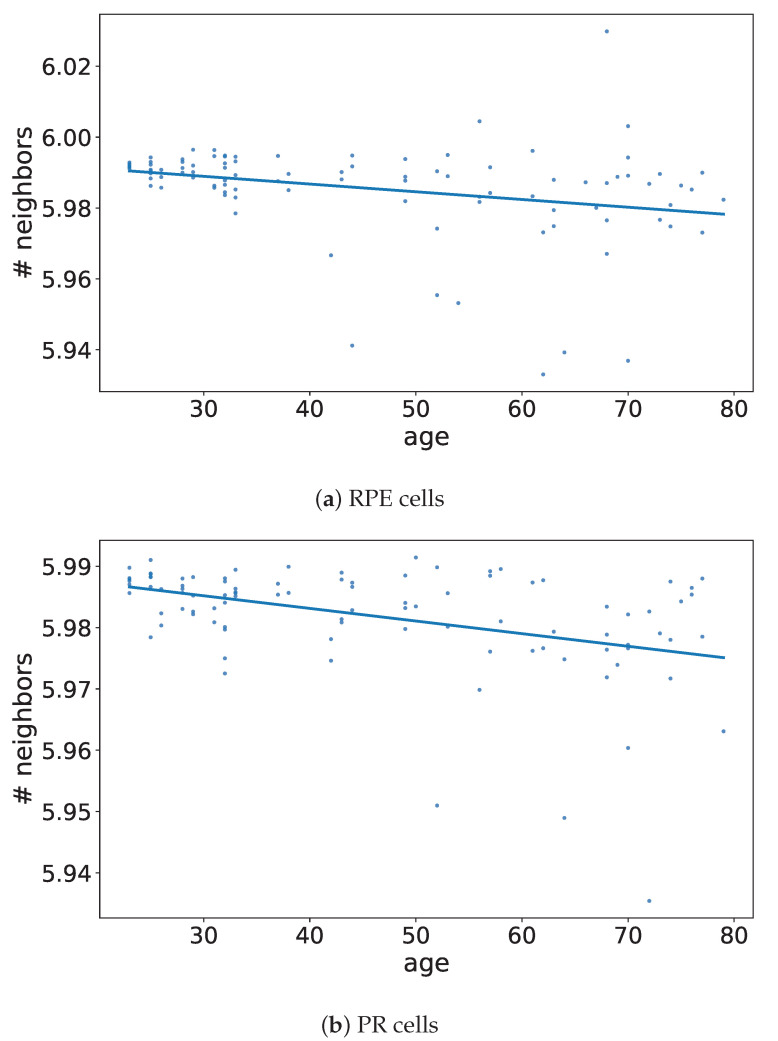
Number of neighbors in Voronoi tessellation cells as a function of age for (**a**) RPE cells and (**b**) PR cells.

## Data Availability

As this is a clinical trial, the data cannot be shared openly. Upon request to the corresponding author S.F., the dataset and code for the statistical analysis can be made available for review.

## Abbreviations

The following abbreviations are used in this manuscript:

RPE	Retinal Pigment Epithelium
PR	Photoreceptor
AO	Adaptive Optics
AO-FI	Adaptive Optics Flood Illumination
AO-TFI	Adaptive Optics Transscleral Flood Illumination
AMD	Age-Related Macular degeneration
DR	Diabetic Retinopathy
ML	Machine Learning
OCT	Optical Coherence Tomography

## Appendix A

Here we present supplementary material to the main results of Section 3.
